# Distinct Expression Pattern of a Deafness Gene, *KIAA1199*, in a Primate Cochlea

**DOI:** 10.1155/2016/1781894

**Published:** 2016-06-14

**Authors:** Makoto Hosoya, Masato Fujioka, Hideyuki Okano, Kaoru Ogawa

**Affiliations:** ^1^Department of Otorhinolaryngology, Head and Neck Surgery, Keio University School of Medicine, 35 Shinanomachi, Shinjuku-ku, Tokyo 160-8582, Japan; ^2^Department of Physiology, Keio University School of Medicine, 35 Shinanomachi, Shinjuku-ku, Tokyo 160-8582, Japan

## Abstract

Deafness is one of the most common types of congenital impairments, and at least half of the cases are caused by hereditary mutations. Mutations of the gene* KIAA1199* are associated with progressive hearing loss. Its expression is abundant in human cochlea, but interestingly the spatial expression patterns are different between mouse and rat cochleae; the pattern in humans has not been fully investigated. We performed immunohistochemical analysis of a nonhuman primate, common marmoset (*Callithrix jacchus*), cochlea with a KIAA1199-specific antibody. In the common marmoset cochlea, KIAA1199 protein expression was more widespread than in rodents, with all epithelial cells, including hair cells, expressing KIAA1199. Our results suggest that the primate pattern of KIAA1199 expression is wider in comparison with rodents and may play an essential role in the maintenance of cochlear epithelial cells.

## 1. Introduction

Deafness is one of the most common types of congenital impairment, and at least half of all cases are caused by hereditary mutations. In some patients, hearing loss is progressive, with hearing loss developing gradually during childhood or youth after the acquisition of speech abilities. Mutation of the* KIAA1199* gene is one cause of hereditary hearing loss [1].

*KIAA* genes were identified by sequence analysis of human large (>4 kb) cDNAs in the Kazusa cDNA sequencing project [2].* KIAA1199* was found in a sequence of cDNA clones of unknown human genes from human adult and fetal brain cDNA libraries in 1999 [3].* KIAA1199* was later identified by cDNA microarray analysis as a cochlea-specific gene that was abundantly expressed in the human cochlea [4], and the protein it encodes has been found to play a central role in hyaluronan binding and depolymerization [5]. Recently, this gene became of interest in the cancer research field after clinical studies found it to have an involvement in cancer progression, metastasis, and poor prognosis of patients [6].

Signal cascade analyses revealed that* KIAA1199* is a likely target gene of the Wnt/*β*-catenin signaling pathway [7], which is also known to be a key pathway in inner ear development [8] and regeneration [9]. Mutations of* KIAA1199* cause progressive hearing loss with a downsloping pattern, and usually the hearing impairment starts after acquisition of languages [1]. In such postlingual hearing loss, in general, the auditory cortex has already developed and prevention of progressive hearing loss in the inner ear would be expected to be the most promising therapy for retaining long-term hearing ability; however, there is currently no such effective treatment for this condition. Thus, understanding the physiological functions of* KIAA1199* and its pathophysiology when mutated is an important issue.

Transgenic or knockout animal models are powerful tools for clarifying disease mechanisms. In many genetic disorders, including hereditary hearing loss, their mechanisms have been unveiled by using animal models, especially transgenic or knockout mouse models [10]. So far, no animal model harboring* KIAA1199* mutations or its knockout has been reported. Expression analysis of KIAA1199 protein in the cochlea has been performed in mice and rats [1, 11], where different distribution patterns for each species were described, suggesting the possibility of an even greater difference in primates. We therefore examined expression of KIAA1199 protein by immunohistochemistry in cochlea from a nonhuman primate, the common marmoset (*Callithrix jacchus*).

## 2. Materials and Methods

### 2.1. Specimens of the Common Marmoset

Fixed and decapitated cadaverous heads of newborn common marmoset (postnatal day 2) and 3–6-year-old common marmosets were kindly provided by Junichi Hata, Reona Kobayashi, Takahiro Kondo, Kimika Yoshino-Saito, and Seiji Shiozawa. Fixed skin samples were also kindly provided. The animal experiments were approved by the Ethics Committee of Keio University (number 11006) and were in accordance with the guidelines of the National Institutes of Health and the Ministry of Education, Culture, Sports, Science, and Technology of Japan.

### 2.2. Tissue Preparation

Temporal bones from newborn and young adult marmosets were dissected, fixed, decalcified with Decalcifying Solution B (Wako, Saitama, Japan) for 3-4 weeks, and embedded in Tissue-Tek OCT compound (Sakura Finetek, Tokyo, Japan) for cryosections. The 7 *μ*m sections were used for immunohistochemistry. Skin samples from young adult marmosets were dissected and fixed.

Temporal bones from 3-week-old mice (C57BL/6) and 3-month-old rats were dissected, fixed, decalcified with Decalcifying Solution B for 3–10 days, and embedded in Tissue-Tek OCT compound for cryosection. The 7 *μ*m sections were used for immunohistochemistry. These animal experiments using mice were approved by the Ethics Committee of Keio University (number 08020) and were in accordance with the guidelines of the National Institutes of Health and the Ministry of Education, Culture, Sports, Science and Technology of Japan.

### 2.3. Immunohistochemistry

After a brief wash with phosphate-buffered saline (PBS) the sections were heated (80°C) in 10 mM citrate buffer (pH 6) for 1 h. After another brief wash, the sections were preblocked for 1 h at room temperature with 10% normal serum in PBS, incubated with primary antibodies at 4°C overnight, and then incubated with Alexa Fluor-conjugated secondary antibodies (Alexa488, Alexa555, and Alexa647) for 60 min at room temperature. The nuclei were counterstained with Hoechst 33342.

### 2.4. Whole-Mount Immunofluorescence

After 6 weeks of decalcifications of the cochlea of common marmoset, sensory epithelium containing organ of Corti was dissected. Sections were incubated with 10% normal serum in PBS for 1 h at room temperature and then with primary antibodies at 4°C overnight, followed by an incubation with Alexa Fluor-conjugated secondary antibodies (Alexa555 and Alexa647) and Phalloidin-conjugated Alexa488 (Thermo Fisher Scientific) for 60 min at room temperature. The nuclei were counterstained with Hoechst 33342.

### 2.5. Antibodies

The primary antibodies used in this study are as follows: anti-KIAA1199 (rabbit immunoglobulin G (IgG), Proteintech, Manchester, UK, 21129-1-AP, 1 : 50, rabbit IgG, Cosmo Bio, Tokyo, Japan, CNP-IP-208, 1 : 50), anti-SOX2 (goat IgG, Santa Cruz Biotechnology, Dallas, TX, US, sc17320, 1 : 100), anti-CTGF (goat IgG, Santa Cruz Biotechnology, sc14939, 1 : 100), anti-CALDESMON (Sigma-Aldrich, C0297, 1 : 100), anti-NKCC1 (goat IgG, Santa Cruz Biotechnology, sc21545, 1 : 300), anti-MYOSIN7a (mouse IgG, DSHB, Iowa City, IA, US, 138-1-s, 1 : 30), and anti-*β*-III TUBULIN (mouse IgG, Sigma-Aldrich, T8660, 1 : 250).

## 3. Results and Discussion

Recently we reported a brief document about the basic morphology and feasible methods for immunohistochemical analysis of the cochlea of the common marmoset (*Callithrix jacchus*) [12], which is a useful animal in modeling human disease by generating transgenic monkeys [13]. The cochlea of marmosets has high similarity with that of humans in its basic morphology and protein distributions as defined by immunohistochemistry. Our previous results identified discrepancies in protein expression patterns in the cochlea between primates and rodents, indicating the necessity of studying primates, especially in regard to the deafness genes, where the typical human symptoms cannot be reproduced in mouse models; for example, see* CX31* [14] and* CRYM* [15].

**Figure 1:**
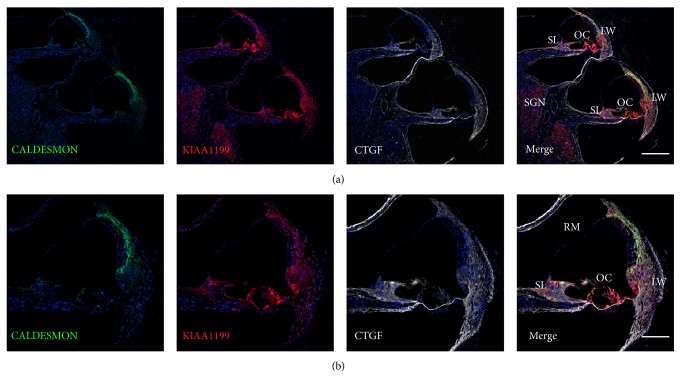
Expression of KIAA1199 in the cochlea of the common marmoset. (a and b) KIAA1199 expression is observed in the lateral wall of the cochlea, sensory epithelium, spiral limbus, and spiral ganglion neuron. No expression is observed in Reissner's membrane. LW: lateral wall of cochlea, OC: organ of Corti, SL: spiral limbus, SGN: spiral ganglion neuron, and RM: Reissner's membrane. The nuclei were counterstained with Hoechst (blue). Scale bar: 200 *μ*m in (a) and 100 *μ*m in (b).

In the common marmoset, KIAA1199 protein expression is observed in the lateral wall spiral ligament, hair cells, supporting cells, spiral limbus, and spiral ganglion neurons (Figure 1). No immunoreactivity was observed in Reissner's membrane or beneath the basilar membrane.

**Figure 2:**
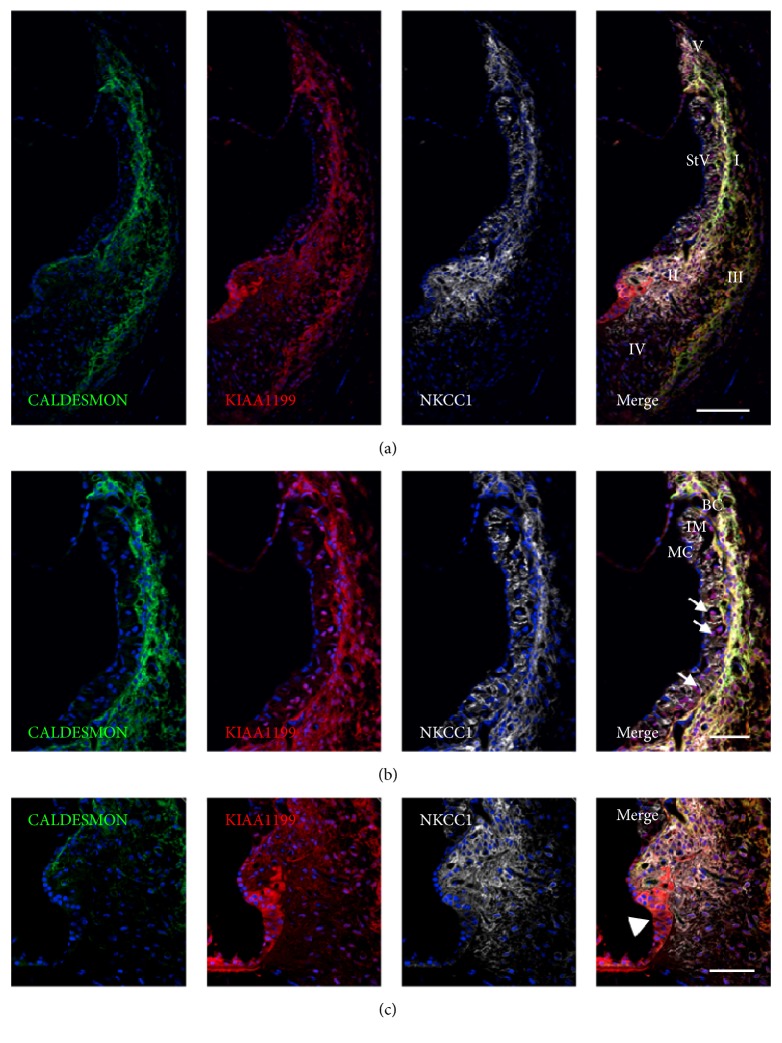
Expression of KIAA1199 in the lateral wall. (a) KIAA1199 expression is observed in type I, II, III, and V spiral fibrocytes. (b) KIAA1199 expression is observed in intermediate cells (arrow) and basal cells in the stria vascularis. No expression is observed in NKCC1 positive marginal cells. (c) KIAA1199 expression is markedly observed in outer sulcus cells (arrow head). No expression was observed in type IV spiral fibrocytes. StV: stria vascularis, I–V: type I–V spiral ligament fibrocytes, MC: marginal cells, IM: intermediate cells, and BC: basal cells. The nuclei were counterstained with Hoechst (blue). Scale bar: 100 *μ*m in (a) and 50 *μ*m in (b) and (c).

In the spiral ligament, KIAA1199 expression was observed in type I, II, III, and V fibrocytes (Figure 2(a)). In the stria vascularis, KIAA1199 expression was observed in intermediate cells and basal cells, whereas no immunoreactivity was observed in NKCC1 positive marginal cells (Figure 2(b)). Notably, KIAA1199 immunoreactivity was relatively strong in the outer sulcus cell (Figure 2(c)).

**Figure 3:**
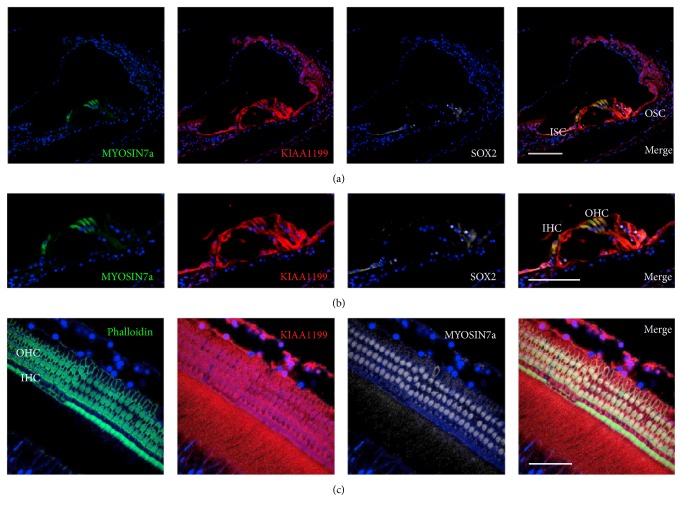
Expression of KIAA1199 in the organ of Corti. (a) KIAA1199 expression is observed in the organ of Corti. (b) KIAA1199 expression is observed in MYOSIN7a-positive hair cells and supporting cells between the outer and inner sulcus cells, including SOX2-positive supporting cells. (c) Whole-mount immunofluorescence also showed broad expressions of KIAA1199 in the organ of Corti. ISC: inner sulcus cells, OSC: outer sulcus cells, IHC: inner hair cells, and OHC: outer hair cells. The nuclei were counterstained with Hoechst (blue). Scale bar: (a) and (b): 100 *μ*m, (c): 50 *μ*m.

**Figure 4:**
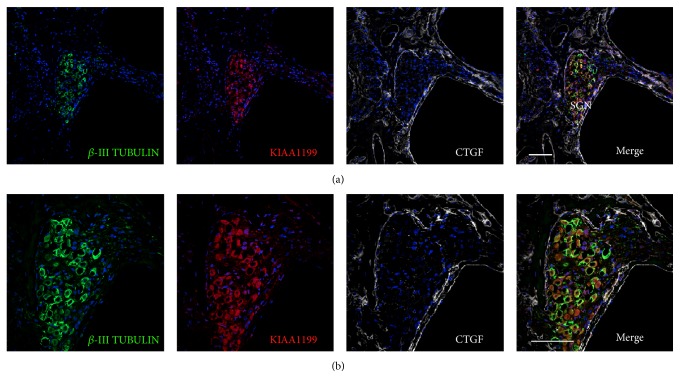
Expression of KIAA1199 in the spiral ganglion neurons. (a and b) KIAA1199 expression is observed in the spiral ganglion neurons. KIAA1199 expression is observed in *β*-III tubulin positive spiral ganglion neurons (SGN). The nuclei were counterstained with Hoechst (blue). Scale bar: 100 *μ*m.

In the organ of Corti, KIAA1199 expression was broadly observed in supporting cells between the inner and outer sulcus cells as well as the inner and outer hair cells (Figure 3). In the spiral ganglion neurons, KIAA1199 expression was observed in *β*-III tubulin positive neurons (Figure 4).

**Figure 5:**
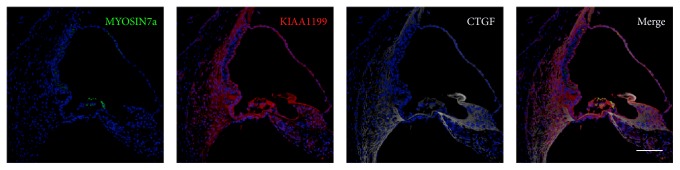
Expression of KIAA1199 in the newborn common marmoset. KIAA1199 expression is observed in the cochlea of newborn common marmoset (postnatal day 2). Immunoreactivities of KIAA1199 in the cochlear epithelial cells including hair cells, supporting cells, and outer sulcus cells were observed. Expressions were also detected in the lateral wall fibrocytes and stria vascularis. The nuclei were counterstained with Hoechst (blue). Scale bar: 100 *μ*m.

We also examined the expression of KIAA in the newborn of common marmoset. Immunoreactivities for KIAA1199 were observed in the lateral wall spiral ligament, hair cells, supporting cells, spiral limbus, and spiral ganglion neurons (Figure 5). The result indicates that this KIAA1199 expression pattern is maintained during their lifetime.

**Figure 6:**
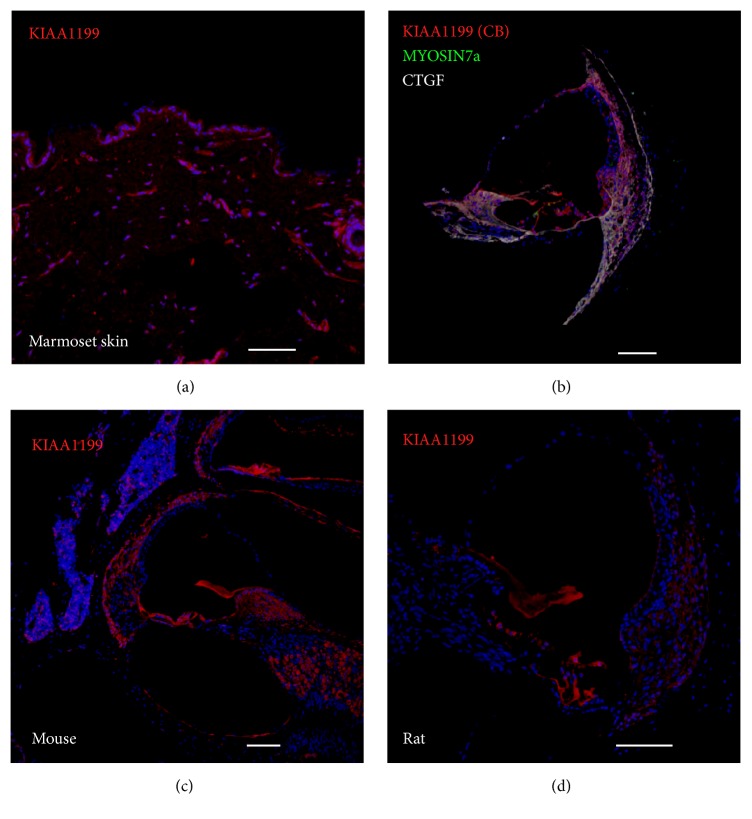
Validations of anti-KIAA1199 antibodies used in this study. (a) KIAA1199 expression is detected in the skin of the common marmoset. (b) Another anti-KIAA1199 antibody (Cosmo Bio: CB) revealed the same expression patterns of KIAA1199 in the common marmoset cochlea. (c) Immunohistochemistry with the cochleae of mouse. The immune reactivities in the lateral wall fibrocytes and spiral limbus were detected as reported previously. (d) Immunohistochemistry with the cochleae of rat. The immune reactivities were restricted in the supporting cells as reported previously. The nuclei were counterstained with Hoechst (blue). Scale bar: 100 *μ*m.

To validate the anti-KIAA1199 antibody (Proteintech) used in this study, we performed immunostaining in the skin of the common marmoset where both immunostaining and in situ hybridization were previously performed [16] (Figure 6(a)). Our results using the skin of common marmoset completely agreed with the previous reports of KIAA1199 expression in the skin of human. Next, we used another anti-KIAA1199 antibody (Cosmo Bio) to reconfirm our observations. The distribution patterns of the immunoreactivity for KIAA1199 using this other antibody was observed (Figure 6(b)). Furthermore, we stained the cochleae of mouse and rat with the antibodies used in this study (Figures 6(c) and 6(d)). In the mouse cochlea, immunoreactivities were detected in the lateral wall fibrocytes and the spiral limbus as previously reported [1]. Of note, the presenting staining results in the rat cochlea with anti-KIAA1199 antibody completely matched those reported in a previous report [11].

The expression of KIAA1199 in the common marmoset was more widespread than that in the mouse or rat. Expression of KIAA1199 has been reported in the fibrocytes of the spiral ligament and the spiral limbus with a transient expression in Deiter's cells at P0 in the mouse [1] (Table 1). In the rat, KIAA1199 expression was observed in supporting cells; however, its expression was not observed in hair cells and outer sulcus cells where obvious expression was detected in the common marmoset cochlea. In short, there is a large species difference in the pattern of KIAA1199 expression in the cochlea across mice, rats, and primates.

To understand the pathophysiology of a progressive hereditary hearing loss in patients, it is necessary to understand the roles of the deafness gene in the maintenance of hearing. Recently, in cancer research, a relationship between the Wnt/*β*-catenin signaling pathway and KIAA1199 was reported [6]. KIAA1199 regulates the Wnt/*β*-catenin signaling pathway by interacting with ASCL2, LGR5, ITPR3, and Ca^2+^ signaling [6, 17, 18]. In the inner ear, Wnt/*β*-catenin signals are essential for the morphogenesis and regeneration of sensory and nonsensory epithelial cells [9, 19, 20]. The expression of KIAA1199 in the sensory epithelium in the marmoset, where Wnt activation is essential for its proliferation, may allow it to interact with the signal cascade and play an important role in cellular maintenance, as seen in cancer cells.

In a future study, functional analysis of KIAA1199 in the inner ear is necessary for understanding* KIAA1199*-related deafness. In such experiments, in general, generating transgenic or knockout mice is the first step. However, for KIAA1199, our observation showed large discrepancies of expression patterns in the cochlea between rodents and primates. Therefore, it is likely that a mouse model will fail to reproduce the hearing impairment observed in human patients. Thus, for an* in vivo* animal model, generating a transgenic primate model, such as a common marmoset, would be required.

## 4. Conclusion

KIAA1199 showed a primate-specific expression pattern in the cochlea. Future functional as well as mutation screening studies using primates will be crucial to understanding the mechanisms of* KIAA1199*-related hearing loss.

## Figures and Tables

**Table 1:** Differential expression of KIAA1199 in marmosets and rodents.

	Expression patterns														Ref.
	Hair cells		Supporting cells												
	Inner hair cell	Outer hair cell	Inner sulcus cells	Inner pillar cells	Outer pillar cells	Deiter's cells	Hensen cells	Claudius cells	Outer sulcus cells	Spiral ligament	Spiral limbus	Stria vascularis	Reissner's membrane	SGN	
Mouse	−	−	−	−	−	+ (~P7)	−	−	−	+	+	−	−	−	Abe et al. 2003 [1]
Rat	−	−	−	+	+	+	−	−	−	NA	NA	NA	NA	NA	Usami et al. 2008 [11]
Marmoset	+	+	+	+	+	+	+	+	+	+	+	+	−	+	
